# Molecular docking analysis of docetaxel analogues as duel lipocalin 2 inhibitors

**DOI:** 10.6026/97320630016438

**Published:** 2020-06-30

**Authors:** Rajagopal Ponnulakshmi, Umapathy Vidhya Rekha, Ramakrishnan Padmini, Srinivasan Perumal, Radhakrishnan Saravanan, Veeraraghavan Vishnupriya, Periyasamy Vijayalakshmi, Jayaraman Selvaraj

**Affiliations:** 1 Central Research Laboratory, Meenakshi Academy of Higher Education and Research (Deemed to be University), Chennai-600 078, India; 2 Department of Public Health Dentistry, Sree Balaji Dental College and Hospital, Pallikaranai, Chennai-600 100, India; 3 Department of Biochemistry, School of life science, Vels Institute of Science, Technology and Advanced Studies (VISTAS), Chennai-117, India; 4 Department of Biochemistry, Karpaga Vinayaga Institute of Dental Sciences, Madhuranthagam, Chengalpattu District, Tamil Nadu, India; 5 Department of Biochemistry, Saveetha Dental College and Hospitals, Saveetha Institute of Medical and Technical Sciences, Saveetha University, Chennai - 600 077, India; 6 PG and Research Department of Biotechnology and Bioinformatics, Holy Cross College (Autonomous), Trichy- 620002, Tamil Nadu, India

**Keywords:** Lipocalin 2, docetaxel, analogues, molecular docking

## Abstract

Lipocalin 2 (Lcn2, also called as neutrophil gelatinase-associated lipocalin) is a member of the lipocalin family and a known target for breast cancer. Therefore, it is of interest to use Docetaxel as a scaffold to design molecules with improved efficiency from naturally derived phytochemicals. We document 10 analogues (4Deacetyltaxol, 7Acetyltaxol, Cabazitaxel, Cephalomannine, Docetaxal, Deacetyltaxol, Docetaxeltrihydrate, Ortataxel, Paclitaxel, Taxoline) having optimal binding with Lipocalin 2 in comparison with Docetaxel. This data is highly useful for consideration in the design and development of drugs for breast cancer.

## Background

**Figure 1:**
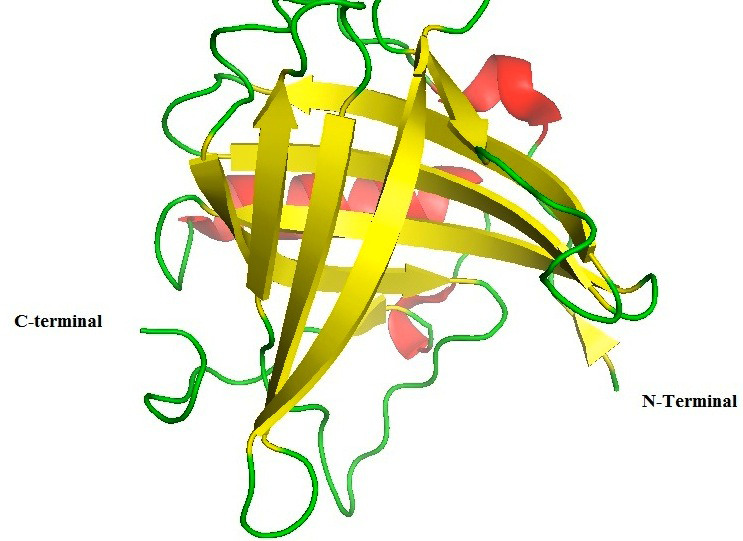
Structure of lipocalin 2

Breast cancer is an issue of medical importance worldwide [1-3]. Treatments such as radiation therapy, chemotherapy, surgery, immunotherapy, and hormone therapy are available with debatable efficiency. Known drugs in this context is under constant debate for efficiency and drug resistance [4,5]. The use of an FDA approved drug docetaxel as a therapeutic agent in cancer patients are known [6-10]. Lipocalin 2 (Lcn2, neutrophil gelatinase-associated lipocalin (Figure 1) is a member of the lipocalin family and a known target for breast cancer [11-18]. Therefore, it is of interest to use Docetaxel as a scaffold to design molecules with improved efficiency from naturally derived phytochemicals.

## Methods:

### Protein preparation:

The X-ray crystallographic structure of the lipocalin 2 with 2.6Å resolution was retrieved from Protein Data Bank (PDB) with PDB ID: 1DFV was used in this study using standard procedure [19].

### Ligand preparation:

Structure of Docetaxel and its 10 analogues were downloaded from the PUBCHEM database in SDF format and converted to PDF file format with the help of the Online Smile Translator.

### Molecular docking analysis:

Molecular docking analysis was completed using PATCHDOCK following standard protocols [20,21]. The docked structure was examined using Ligplot [22].

## Results and discussion:

**Figure 2:**
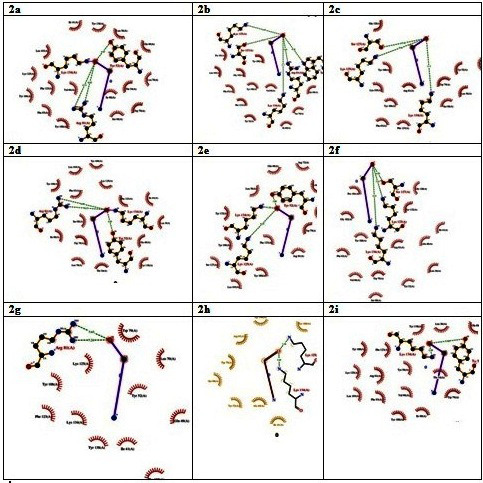
Ligplot analysis of docked complex showing interaction of lipocalin 2 with (a) 4Deacetyltaxol; (b) 7Acetyltaxol; (c) cabazitaxel; (d) Cephalomannine; (e) Docetaxal; (f) Deacetyltaxol; (g) Docetaxeltrihydrate; (h) ortataxel; (i) paclitaxel; (j) taxoline

Table 1 shows the Molecular docking analysis of Docetaxel analogues as duel Lipocalin 2 inhibitors. We document 10 analogues (4Deacetyltaxol, 7Acetyltaxol, Cabazitaxel, Cephalomannine, Docetaxal, Deacetyltaxol, Docetaxeltrihydrate, Ortataxel, Paclitaxel, Taxoline) with desirable binding with the Lipocalin 2 in comparison with Docetaxel (Table 1). Results of the analogue deacetyltaxol have the good binding energy (-132-89 kcal/mol). Figure 2 shows ligand-protein interaction drawn using LigPlot. The interacting residues with optimal hydrogen bonding patterns are shown. An increased amount of hydrophobic atoms in the active center of drug–target boundary enlarged the biological action of the lead [23].

## Conclusions:

We document 10 analogues (4-deacetyltaxol, 7-acetyltaxol, cabazitaxel, cephalomannine, docetaxal, deacetyltaxol, docetaxeltrihydrate, ortataxel, paclitaxel and taxoline) with desirable binding features with the Lipocalin 2 in comparison with Docetaxel for further in vivo and in vitro validation.

## Figures and Tables

**Table 1:** Molecular docking analysis of docetaxel analogues as duel lipocalin 2 inhibitors

S. No	Compound name	Score	ACE	Atomic interaction	Ligand atom	Distance	No of non bonded interaction
1	Docetaxel	5804	-54.82	LYS 125	NZ-O	1.53	57
				LYS 134		3.02	
1	4Deacetyltaxol	6474	-147.98	TYR 52	OH-O	2.87	117
				ARG 81	NH-0	1.49	
				LYS 134	NZ-O	3.32	
2	7Acetyltaxol	6252	-103.92	TRP 79	NE-O	2.3	114
				ARG 81	NH2-O	3.29	
				LYS 125	NZ-O	2.83	
				SER 127	OG-O	1.39	
				LYS 134	NZ-O	3.14	
3	Cabazitaxel	5952	-50.11	LYS 125	NZ-O	2.24	69
				SER 127	OG-O	2.44	
				LYS 134	NZ-O	3.29	
				LYS 34	NZ-O	3.03	
4	Cephalomannine	6794	-113.10	TYR 52	OH-O	2.62	110
				ARG 81	NH1-O	1.43	
				ARG 81	NH2-O	2.17	
				LYS 134	NZ-O	1.84	
5	Docetaxal	6404	-111.73	TYR 52	OH-O	3	108
				TYR 52	OH-O	2.81	
				LYS 125	NZ-O	3.28	
				LYS 134	NZ-O	2.63	
6	Deacetyltaxol	5694	-132.89	LYS 125	NZ-O	3.04	87
				SER 127	OG-O	2.68	
				LYS 134	NZ-O	2.05	
7	Docetaxeltrihydrate	6022	-63.23	ARG 81	NH1-O	2.39	84
				ARG 81	NH2-O	1.34	
8	Ortataxel	6204	-55.51	LYS 125	NZ-O	2.26	74
				LYS 134	NZ-O	3.05	
9	Paclitaxel	6438	-121.39	TYR 52	OH-O	2.4	148
				LYS 134	NZ-O	2.43	
10	Taxoline	6824	-74.36	TYR 52	OH-O	2.37	83
				ARG 81	NH1-O	2.78	
				ARG 81	NH2-O	2.94	
				LYS 125	NZ-O	2.87	
				LYS 134	NZ-O	2.65
